# Morphological Description of Frontal EEG Interictal and Ictal Discharges in an Adult Cohort of 175 Patients

**DOI:** 10.3390/jcm10061219

**Published:** 2021-03-15

**Authors:** Beatriz García-López, María Sueiras-Gil, Ana Isabel Gómez-Menéndez, Fernando Vázquez-Sánchez, María Carmen Lloria-Gil, Jerónimo J. González-Bernal, Josefa González-Santos, Mirian Santamaría-Pelaéz, Raúl Soto-Cámara, Troels Wesenberg Kjaer

**Affiliations:** 1 Neurophysiology Department, Burgos University Hospital, 09006 Burgos, Spain; bgarcialo@saludcastillayleon.es (B.G.-L.); agomm@saludcastillayleon.es (A.I.G.-M.); mclloriag@saludcastillayleon.es (M.C.L.-G.); 2 Neurophysiology Department, Valld’Hebron University Hospital, 08035 Barcelona, Spain; msueiras@vhebron.net; 3 Neurology Department, Burgos University Hospital, 09006 Burgos, Spain; 4 Department of Health Sciences, University of Burgos, 09001 Burgos, Spain; jejavier@ubu.es (J.J.G.-B.); mspelaez@ubu.es (M.S.-P.); 5 Sjællands Universitets Hospital, 4000 Roskilde, Denmark; twk@regionsjaelland.dk

**Keywords:** EEG, frontal lobe seizures, electroencephalography, EEG discharges in focal epilepsy, EEG morphology

## Abstract

Clinical and electroencephalogram (EEG) features in frontal lobe epilepsy (FLE) vary considerably among patients, making the diagnosis a challenge. The objective of this study was to describe interictal and ictal EEG activity, identifying variables that could help to differentiate and diagnose frontal lobe epilepsy cases. A prospective cross-sectional study from patients with frontal interictal epileptiform discharges (IED) referred to the Vall d’Hebron University Hospital (Barcelona, Spain) after a clinical event compatible with epileptic seizures was designed. The interictal and ictal activity were analyzed to provide a detailed EEG description of the cases, using different statistical analyses. The morphological seizure pattern at the ictal onset remained globally unchanged over time in seizures arising from the frontal lobe for each patient. Isolated sharp waves were the most frequent waveforms in the expression of IED. Frontal lobe seizures are frequently short and sometimes appear grouped in clusters within the same recording. Often the ictal expression of the electrical activity in frontal lobe seizure is subtle and challenging to interpret. A description of the main findings is summarized to identify seizures arising from the frontal lobe and avoid false negatives findings in EEG interpretations.

## 1. Introduction

Frontal lobe epilepsy (FLE) is the second most frequent type of focal epilepsy. Most series are surgical, which may introduce selection bias to pharmaco-resistant focal epilepsy as less than 1% of these patients undergo surgery [1,2,3].

The International League Against Epilepsy (ILAE) classifies FLE into seven subtypes regarding the location of seizure origin: primary sensorimotor cortex, supplementary sensorimotor cortex, orbit-frontal cortex, frontopolar cortex, dorsolateral frontal cortex, cingulate cortex, frontoparietal operculum [4].

Seizures arising from the frontal lobe can easily be misdiagnosed due to their very variable clinical manifestations [1,5]. They often present during sleep, sometimes in clusters, frequently with early motor symptoms, none of them specific (1), which makes clinical classification more difficult [6,7].

Interictal epileptiform discharges (IED) appear in most patients with FLE as electroencephalogram (EEG) epileptiform discharges arising from an epileptic focus and usually spreading beyond the frontal regions [8]. EEG abnormalities are sometimes subtle, and muscle artifacts often obscure the ictal recordings. Regarding ictal activity, bilateral paroxysmal EEG discharges appear with an amplitude asymmetry and often preceded by a bilateral electro-decremental activity, representing secondary synchrony rather than actual generalized seizure onset [4]. All these facts make EEG very difficult to read.

Most studies on EEG of FLE focus on spike frequency or seizure patterns rather than the added value of considering several characteristics simultaneously. 

The main objective was to describe interictal and ictal EEG activity, identifying variables that can help to differentiate and diagnose frontal lobe epilepsy cases. Minor objectives are to study if there is a significant relationship between the pathologic antecedents and focus location, between the location of seizure onset and the IED location and the morphological aspect, and whether the morphology of the seizure pattern remains the same over time for each patient.

## 2. Materials and Methods

### 2.1. Study Design and Participants

This was a prospective cross-sectional study with 175 patients from the Electroencephalography Section of the Clinical Neurophysiology Department at the Vall d’Hebron University Hospital, Barcelona (Spain). The inclusion criteria were EEG with frontal region interictal or ictal epileptiform discharges in patients with previous diagnosis of frontal lobe epilepsy or patients that fulfill the criteria but were not yet diagnosed as they were still being studied (one or more episode clinically suggestive of frontal lobe seizures and EEG supportive, but still no formal diagnosis, as they were waiting for the referral neurologist to review the result of the EEG and MRI). 

Clinically, the symptomatology suggestive of frontal lobe seizure is understood as the presence of one or more of the following manifestations: focal motor manifestations, fencer posture, “sign of four” posture, loud vocalizations, focal atonic features, hyper-motor manifestations, “chapeau de gendarme”, unmotivated laugh (gelastic seizures), axial tonic-clonic movements, forced head or eye deviation, atonia, tonic manifestation, autonomic manifestations, aphasia or dysphasia, automatisms at seizure onset, impaired awareness, olfactory hallucinations and illusions [4].

Cases with more amplitude in the mid-temporal areas were excluded in order to avoid the selection of temporal focus cases.

The study received a favorable report from the Institutional Review Board (Burgos University Hospital-Reference IRB 1543) and was conducted in accordance with the ethical principles of the Declaration of Helsinki. 

### 2.2. Procedure-Main Outcomes

For each patient case, all available EEG recordings, both awake and sleep state, including both routine and sleep deprivation recordings, were considered and reviewed. All were performed with video simultaneously. EEG recordings before 2007 were obtained with an analog Nicolet EEG system, while later EEG recordings were obtained with a Deltamed digital system. With the 10/20 system of EEG recording, four frontal regions were defined: fronto-polar (Fp1, Fp2), inferior frontal (F7, F8), central−frontal (F3, F4), and mid−line−frontal (Fz). Given the fact that inferior frontal electrodes (F7-F8) share recording area with anterior temporal regions, cases with maximal voltage in F7/F8 electrodes, in which the amplitude in mid temporal areas (T3/T4) was higher than in other frontal electrode positions (F3/Fz/F4 or Fp1/Fp2), were excluded. The discharges recorded from F7/F8 inferior frontal were then considered, instead of temporal ones, as other frontal electrodes involvement and had higher amplitude than mid temporal discharges in all cases. This strict selection allowed the study of a typical clinical situation of frontal lobe origin. 

The interictal and ictal activity were analyzed to provide a detailed EEG description of the cases. IEDs were coded with as many data points as necessary for each subject, up to five, when several items appeared. For the study of IED, a classification regarding the morphology, the voltage, and the EEG waveform was created. The IED morphology was classified into spike, simple and dysphasic sharp wave, and poly-spikes. Peak to peak voltage was classified in four different groups regarding voltage: <50 µV, 50–100 µV, 100–200 µV, and >200 µV in an average montage. The EEG activity which followed the IED was classified as isolated, regular slow-wave, or irregular slow-wave. The location of the IED was the electrode with the higher voltage in average montage. In cases with great diffusion of the discharges, epochs with less diffusion of the IED were actively searched, including REM sleep epochs when available.

The relationship between the IED location and the onset of seizures location was assessed. To do this, all the patients with a registered seizure were analyzed. One electrode data point was placed at the dominant ictal onset location and up to two electrode locations for IED. Each seizure was studied to assess the most active electrode at seizure onset. When an electrode could not be assigned to the seizure onset location, left hemisphere or right hemisphere were codified, if recordings allowed for lateralization, or classed as undetermined. Cases were coded as undetermined location of seizure onset in two situations: (1) when the most active channel was not clear enough due to artifacts at seizure onset or by global voltage attenuation, (2) in cases recorded on paper with an analog EEG-polygraph, when the electrode montage was not optimized to assign a specific electrode for seizure onset. When more than one seizure was recorded in a patient, all the seizures were studied. When two or more seizures had a precise electrode seizure onset location, the same electrode at the ictal onset location for each patient was always found; that is to say, when an electrode of onset could be assessed, it did not change for a patient among seizures, so that location was used to assess the relationship between the seizure onset location and IED location. Patients with more than one registered seizure contributed their clearest and demonstrative EEG registered seizure, as long as seizure onset morphological pattern and frequency remained the same among seizures.

**Figure 1:**
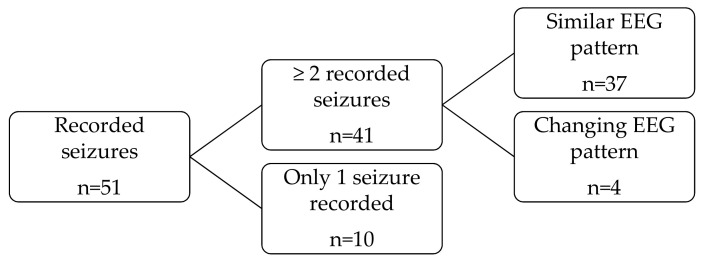
Variability of seizure pattern.

In order to identify if the location, the morphology, the amplitude aspects and whether the onset pattern of the seizures remained the same or changed over time for the same patient, a subgroup with at least two recorded seizures was identified. Regarding morphology of the ictal activity at the onset, the same morphological pattern was assigned when two seizures differed only in amplitude and duration in their evolution. This fact was understood as a consequence of a difference in the discharge intensity rather than a different morphological seizure-onset-pattern (Figure 1). 

Postictal activity and poly-graphic ictal changes, like tachycardia, bradycardia, or irregular breathing, were also taken into account. EEG recordings were analyzed in various montages: in digital recordings, at least two bipolar and an average montage; in analogical recordings, in the available one used for the recording.

Moreover, to perform a detailed and in-depth analysis of the recorded seizure, ictal activity was typified according to morphology and amplitude at ictal onset. Frequencies at onset were studied in the first term. Afterward, EEG onset was analyzed regarding the morphology and amplitude of waveforms during the first two seconds of the ictal discharge to try to categorize the onset of the seizures. 

### 2.3. Statistical Analysis

The categorical variables were summarized as absolute frequencies and percentages, while the continuous variables were expressed in terms of mean and standard deviation (SD). The compliance of the normality criteria of the quantitative variables was assessed by the Kolmogorov-Smirnov test. To evaluate the association between categorical variables, the chi-square test was used; the relationship between categorical and continuous variables was analyzed using the analysis of variance (ANOVA); a Pearson correlation was used to evaluate the association between continuous variables. For the analysis of statistical significance, a value of *p* < 0.005 was established. Statistical analysis was performed with SPSS version 25 software (IBM-Inc., Chicago, IL, USA).

## 3. Results

The number of patients who met the inclusion criteria was 175, contributing to a total of 461 EEG recordings reviewed for this study. The sample was composed of a majority of men over women (54.9% versus 45.1% respectively). The age of the patients at seizure onset ranged from 18 to 93, with a mean of 49 years. This age varied according to the antecedents: the higher mean age was observed in patient cases with vascular or tumor antecedents (42.9 and 41.0 years, respectively); whereas, the lowest was in patients with infectious antecedents (10.5 years) and without any pathological antecedent of interest (22.6 years), and near to the mean age for post-traumatic cases (31 years). The most frequent pathological antecedents were traumatisms (n = 35) or tumors (n = 32), whereas infections (n = 14) was the less common. Frontal lobe lesions were shown in 48.6% of MRI. Any remarkable findings were obtained when the relationship between pathological antecedents and IED morphology was studied.

Clinically, the ictal symptomatology described in clinical records and the EEG recordings were as follows: focal motor manifestations, 27.4%, fencer posture, 2.9%, “sign of four” posture, 2.9%, loud vocalizations, 8.6%, focal atonic features, 24.6%, hyper-motor manifestations, 12%, “chapeau de gendarme”, 5.7%, unmotivated laugh (gelastic seizures), 2.9%, axial tonic-clonic movements, 10.8%, forced head or eye deviation, 22.3%, atonia, tonic manifestation, autonomic manifestations, 23.4%, aphasia or dysphasia, 20%, automatisms at seizure onset, 5.7%, olfactory hallucinations, 2.3%. We found a variable impaired awareness among seizures.

The most common waveform in the overall distribution was isolated sharp wave, presented in 97.1% of the patients, followed by isolated spike in 50.2% of patients, and poly-spikes in 6.9% of the patients. We can see that one patient can present more than one type of IED. Attending to their voltage, apparent differences in the distribution were observed. Sharp waves, simple or dysphasic, were the most common interictal epileptiform morphology for all amplitudes, except for the group of more than 200 µV. The amplitude range of the sharp waves more frequently had a voltage from 50 to 100 µV. In all cases with interictal poly-spike morphology, the amplitudes were less than 100 µV, whereas the voltages of more than 200 µV were found more frequently in the spike group. 

At least one seizure was recorded in 51 out of 175 patients, constituting 28.9% of the cases. Inferior frontal regions were slightly more frequent as the location of ictal onset than the other regions of the frontal lobe. Any difference between the location of seizure onset and morphology at the ictal onset was observed. Despite the lack of association, the seizures arising from prefrontal electrodes did not show any attenuation at onset. There were a relatively high number of cases with undetermined origin (n = 8), in which it was not possible to assess detailed data of seizure onset location. In those cases codified as left hemisphere, right hemisphere or undetermined seizure onset location (n = 18), the IEDs were located in both front-central regions. (Table 1). This fact could mean that IED in front-central regions arose from deeper areas or had greater diffusions, which made both the location of the foci and the location of the seizure onset more complicated. None of the cases had an extra-frontal ictal onset. A statistically significant relationship was observed with a Chi2 test between the location of seizure onset and IED location (*p* = 0.0000).

The beta band was the most common rhythm at seizure onset (n = 30), with a mean frequency of 15.4 Hz, observed in 50.0% of the cases with frontal-central seizures. The alpha band onset was more frequent in seizures arising from the inferior frontal region than in the other frontal areas. There was a dominance of left hemisphere onset with faster frequencies at onset (beta and alpha respect theta and delta); seizures from a left frontal origin (F3) were 42% of all beta band ictal onset. Autonomic manifestations were observed as poly-graphic changes in the chest band and EKG channels in 8% of the recorded seizures. Postictal slowing, with the immediate recovery of alpha rhythm in posterior regions, was observed in some cases. No statistically significant relationship was observed between the location and frequencies at seizure onset.

**Figure 2:**
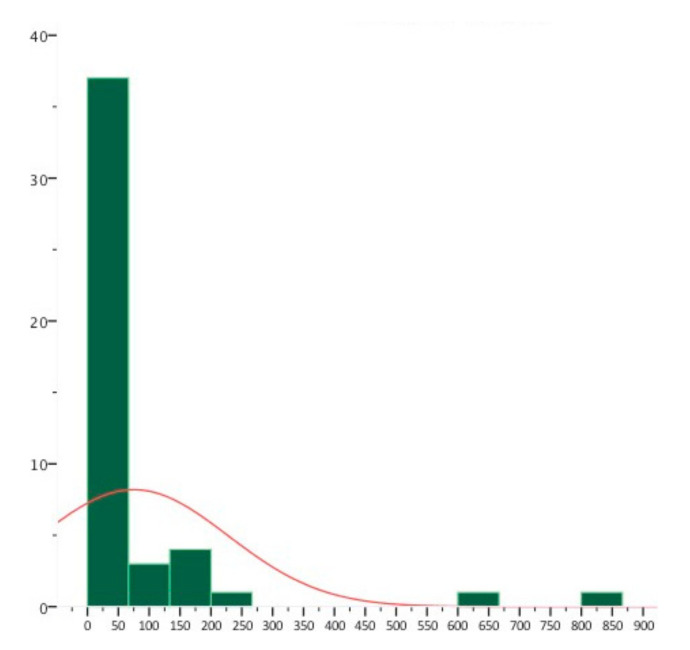
Duration of seizures (x axis, duration of seizures in seconds; y-axis, number of seizures).

The mean duration of seizures was 75 s, having ictal activity as short as 3 s and up to a maximal duration of 15 min. Very brief discharges were considered as ictal when longer seizures with the same morphological pattern with associated clinical manifestations were recorded in the same patient. Recorded seizures were very short, with a median duration of 24 s. A few seizures of a very long duration made the median more accurate than the mean for describing the seizure duration (Figure 2).

According to the EEG morphology and amplitude at seizure onset, rhythmic ictal discharge of >20 µV was the most common category at seizure onset (n = 21), followed by a low amplitude beta ictal rhythm (<20 µV) (n = 14). The location and morphological pattern at ictal onset in EEG with two or more recorded seizures remained the same over time for each patient (*p* < 0.001).

**Figure 3:**
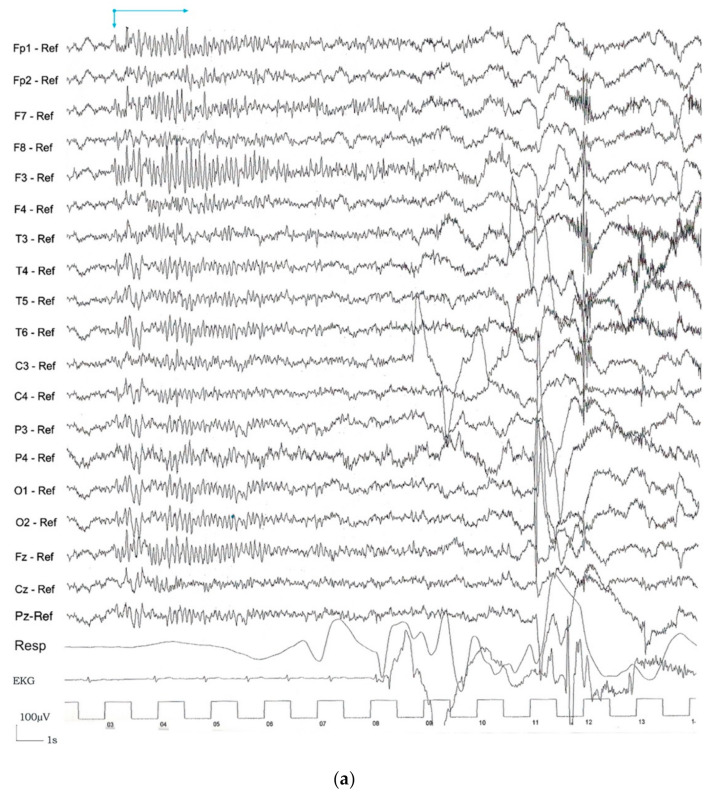

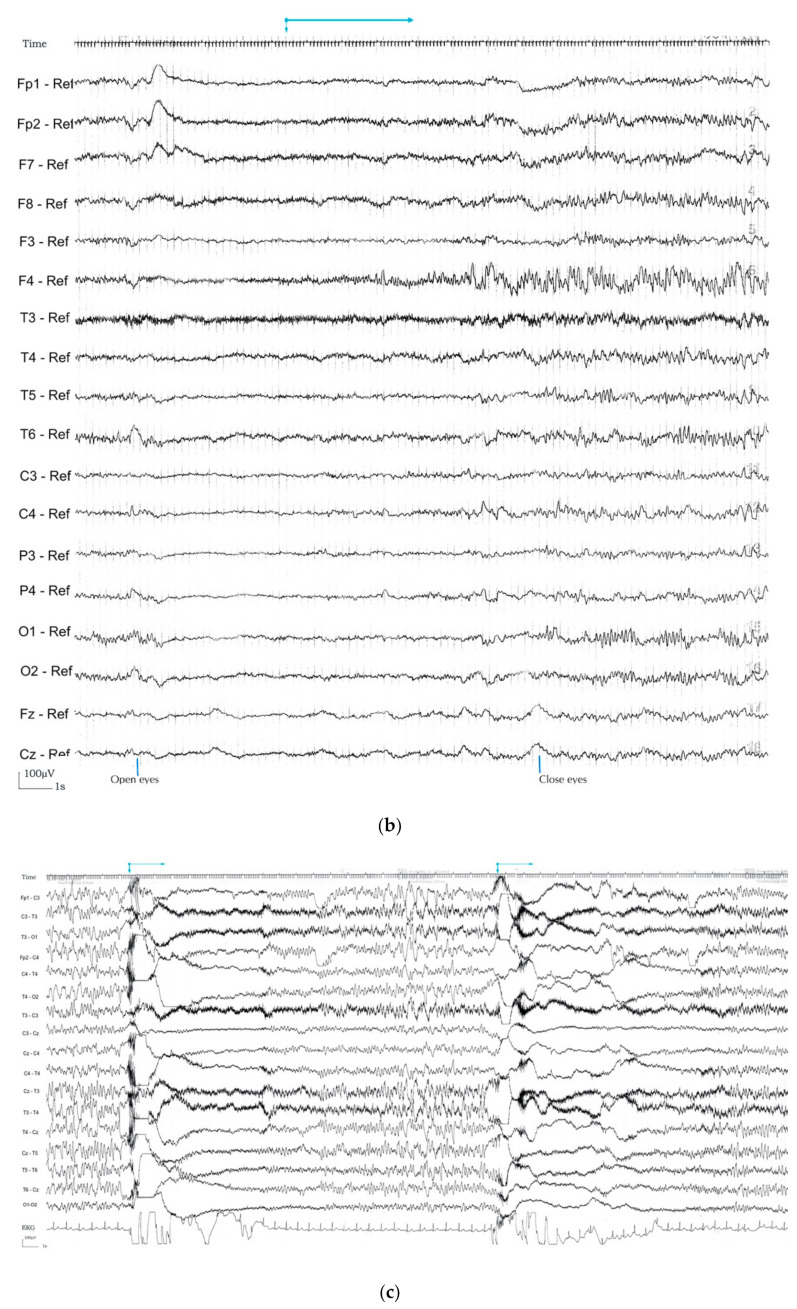

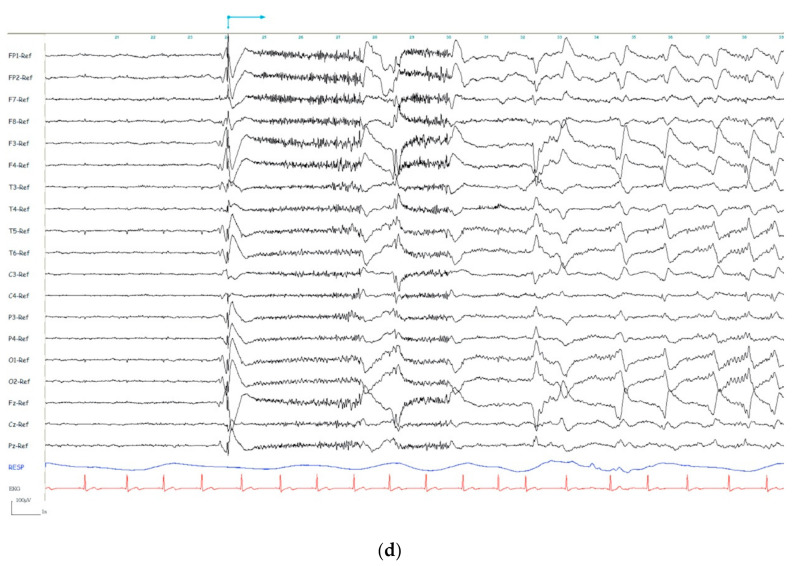
(**a**) Seizure onset patterns: Rhythmic ictal discharge of >20 µV. (**b**) Seizure onset patterns: Low amplitude ictal beta rhythm (<20 µV). (**c**) Seizure onset patterns: Global attenuation (<10 µV). (**d**) Seizure onset patterns: Interictal epileptiform discharge (IED) just prior to the ictal onset.

Regarding the seizure onset, four possible EEG categories for seizure onset pattern were observed: rhythmic ictal discharge of >20 µV (Figure 3a), low amplitude ictal beta rhythm (<20 µV) (Figure 3b), global attenuation (<10 µV) (Figure 3c), and IED just prior to the ictal onset (Figure 3d). The term “global attenuation” was used instead of “generalized attenuation” to avoid nomenclature errors in understanding that these seizures were focal and not generalized. For the description of the ictal onset pattern, all the recorded seizures were studied, and it was observed that all could be assigned to one of the four morphological ictal onset patterns. This category was the same for each patient between seizures.

## 4. Discussion

FLE is the second most frequent type of focal epilepsy, after TLE, representing 20−30% of all focal cases [1,2,9,10]. In recent years, there has been an increasing interest regarding this type of epilepsy. Seizures arising from the frontal lobe are very varied clinically, and their EEG recordings are difficult to read. There is more extensive knowledge of other types of seizures than frontal lobe ones. These differences may be due to the different selection procedures used in various studies. In this sample, they have been found in 88% of symptomatic cases.

This study increases the current neurophysiological knowledge of frontal lobe epilepsy, facilitating the EEG identification of frontal IED and seizures. Combining morphology, amplitude, and frequency of discharges constitutes the novel approach presented here. These aspects are crucial to correctly identify and differentiate frontal lobe from generalized cases.

According to the IED morphology in FLE, a wide variety of interictal waveforms in patients with frontal seizures was found. These include spikes, sharp waves, spikes followed by a slow wave, poly-spikes or poly-spikes followed by a slow wave, and periodic sharp and slow wave complex [10,11]. These findings agree with several studies grouping all IEDs without differentiating among waveforms and correlate with other authors’ work in generalized epilepsies [5,12,13], which in some cases can lead to overlooking meaningful information from an EEG point of view. In this study, the IEDs have been explored and classified, including the voltage and the EEG waveforms following the IED. According to these characteristics, there may be a relation between the type of epilepsy and its EEG features.

IED presents as focal discharges, unilateral discharges over one frontal lobe, multifocal discharges over one frontal lobe, lateralized hemispheric discharges, bi-frontal symmetric, asymmetric, or widespread discharges [6,10,13,14,15]. Regarding widespread interictal epileptiform discharges, Tukel and Jasper demonstrated that focal para-sagittal lesions might show bilateral slow-wave discharges due to secondary bilateral synchrony [16]. Given the broad EEG expression of the epileptiform discharges, it is crucial to carefully evaluate all the EEG recordings’ details to avoid misinterpretation. In this context, the electro-encephalographist must use several montages, with different sensitivity and speed, to get the most out of the recording depending on individual needs.

Even though some authors have previously reported that well−localized frontal foci are the exception [15,17], current technology, with digital EEG recordings, allows for the localization of most foci despite their diffusion. An electrode maximal expression location was assigned to 95% of the foci.

Unspecific irregular slow waves were identified in this sample. Some authors have described other non-epileptiform EEG abnormalities, like slow waves or rhythmical midline theta rhythm. Once excluded, drowsiness and mental activation may give a clue to the abnormal hemisphere, appearing focal or lateralized. Those waves show a slowing or an asymmetry of activity over the two hemispheres [10] and they are an EEG correlation of dysfunction rather than an epileptogenic abnormality [18].

Sleep enhances both epileptic seizures and IED, whereas both are less frequent during REM sleep [19]. Busek et al. found that the activation of cholinergic neurons of the ponto-mesencephalic tegmentum inhibits the thalamus-cortical synchronization during REM sleep, resulting in the suppression of IED and seizures [20]. Epileptiform discharges, facilitated by phasic activity during NREM sleep (spindles and K-complexes), reflect the synchronizing effect of the thalamus-cortical reverberation circuitry [21]. These phasic events are more pronounced over the fronto-central cortex and could explain the finding that seizures arising from the frontal lobe are more frequent during sleep, in contrast to the other brain lobes [22]. The REM sleep-suppression-effect was appreciated in this sample. In cases with a widespread IED, this characteristic is a useful tool to help locate the focus.

A possible relation between etiological causes of FLE and focus location has previously been studied. Frontal lobe tumors have been reported to be more epileptogenic, while traumatic lesions seem to be more frequent in anterior frontal and orbital frontal regions [2]. The pathological antecedents of patients were classified by statistical grouping methods into three different clusters: tumoral and infectious cases related to prefrontal electrodes, vascular antecedents related to inferior frontal focus, and "other" antecedents associated with the left front-central electrode. Nevertheless, these findings are not clinically relevant.

Several authors have identified ictal patterns related to this type of epilepsy, their discharges being quite variable [6,8,12,19,23,24]. These patterns can present as a low-voltage fast activity, as an incrementing or a recruiting rhythm, as a rhythm at different frequencies, as repetitive spikes or spikes and slow waves, as focal or widespread attenuation, as a flattening of the background arrhythmic activity, or as obscure. Depending on the electric dipole position, the seizure may be very difficult to read or even electrically silent with no apparent change evident in the EEG [6,12,19,20,23,25].

The precise location of frontal lobe ictal discharges is often difficult and maybe misleading [9,18,24] (3–5) as the EEG recordings in the sample have shown. The utilization of different available montages increases the possibility of locating the focus and correctly diagnosing the cases.

In contrast to seizures arising from the temporal lobe, the ictal discharges are frequently more widespread and may become evident later in the course of the seizure [10]. Nevertheless, it is recognized that ictal recordings are instrumental in seizure localization and lateralization [25]. The propagation of seizures arising from the frontal lobe to contra-lateral frontal regions and ipsilateral temporal regions causes a problem with localization and the rapid spread of the discharge may obscure its focal onset [17]. Still, a careful study with electronic recording can help to determine the onset location in many cases. This diffuse ictal activity may be a useful morphologic tool to recognize a possible frontal onset.

The analyses of the variability of seizure morphological pattern over time for the same patient has shown that seizures present globally a constant location at onset and the same morphological pattern over time, as reported earlier [3,26,27,28]. 

Foldvary et al. analyzed the localizing value of ictal EEG in focal epilepsy, given a known lesion in both mesial and lateral FLE [24]. They studied seizure specific data like duration, pattern, and distribution of the ictal onset rhythm, finding that repetitive epileptiform activity was significantly more common in lateral frontal lobe epilepsy. In contrast, generalized patterns, defined as activity involving multiple electrodes over both cerebral hemispheres having a less than 2:1 amplitude predominance of one side over the other, were substantially more common in mesial and occipital FLE. Eight patients had entirely obscured seizures, two patients had no EEG changes, and none had rhythmic temporal theta activity. They also referred a low percentage (3%) of mis-lateralized lateral frontal seizures, and 6% of total mis-localized [25]. The higher rate of non-localized seizures was related to the fact that they included in their analysis seizures recorded from all brain lobes. The results of this study, limited to frontal lobe seizures, did not find any remarkable relations and showed 15% of non-localized seizures regarding seizure onset localization.

The findings of this study are in line with several authors in that electroencephalographic ictal onset assessment still plays a major role in localizing the epileptic zone [29].

The short duration of the seizures observed in this sample agrees with previous observations [3,4,19,30]. Manford et al. found that seizures of less than ten seconds with a rapid recovery were more likely to be associated with frontal than temporal lesions [3].

Regarding the relation between the IED location and seizure onset location, 79% of the cases were concordant with an “electrode expression level” (electrode maximal expression of IED to the electrode maximal expression of the ictal onset discharge).This result was as high as 94% when excluding the eleven cases with an unspecific IED location or undetermined seizure onset location. This high percentage might be due to the fact that an amplitude criterion for defining IED was not fixed, and tried to assign focus electrode localization or at least hemisphere localization regardless of its amplitude. Some authors have explored this relation, finding that frontal convexity seizure origin was associated with concordant IED (72% of cases); in contrast, mesial frontal seizures had concordant IED in only 33% of patients [12,30]. In both cases, a minimum amplitude criterion for determining the electrode location was considered.

Epileptic seizures are characterized by a rhythmic activity that progressively increases in amplitude and decreases in frequency. They may develop into a self-sustaining focal epileptic pattern with the capacity to spread across the cortex, precipitating a focal seizure or triggering a generalized seizure [30,31,32,33]. Regarding frequencies at seizure onset, the discharge frequency may vary from 1 to 30/second. Geiger et al. described a transformation from an interictal pattern to a sustained rhythmic pattern in 90% of the studied seizures, with beta frequencies in more than 50% of the seizures [10]. In agreement with these authors, in this sample beta frequencies at onset have been the most frequently observed at seizure onset in seizures arising from the frontal lobe at 58.9%. This finding complements the current knowledge of seizures arising from the frontal lobe, as they included several types of seizures, with only 10/41 cases of frontal origin. According to Worrel et al. [34], a tendency of association between F3 and F4 electrode-position and beta frequencies at onset was observed, especially in left-sided, where all seizures started with beta frequencies. These authors reported a focal beta frequency in 25.9% of patients with frontal convexity seizures. Nevertheless, this percentage was not enough to be significantly associated with convexity location rather than with the frontal lesion’s medial location, a link previously made by some authors [30].

In the sample analyzed, 11.8% of patients presented IED prior to ictal onset, as ictal onset morphology. This pattern had been previously described as a transition from IED to an ictal pattern as a transition sharp wave. Azar et al. have studied this wave as a specific morphological pattern of transition interictal−ictal discharges in neocortical and mesial epilepsy. The pattern was associated with neocortical epilepsy, as none of their patients with mesial epilepsy presented this pattern at onset [35]. They found that 7.1% of all the patients had this pattern at seizure onset. Azar et al. included patients with a presumed hippocampal origin to confirm the absence of a transition sharp-wave pattern in the patients, which may lower the percentage of cases with this morphological pattern at onset in the sample. This pattern has also been studied previously by Ralston and Papetheodorou, who called the ictal discharge after the sharp wave the after-discharge [31,36] Like Geiger and Harner, other authors discouraged the term, pointing to the lack of an obligatory relationship between the sharp discharge and the subsequent rhythmic activity [29]. More recently, Ebersole and Pacia included an ictal onset pattern by runs of periodic sharp waves preceding rhythmic activity [37]. The most recent studies propose that the transition sharp-wave acts as a trigger to the ictal discharge, being sufficient but not necessary to initiate seizures [35]. Avoli et al. have concluded that interictal discharges can prevent ictal discharges, concluding that “interictal spiking is probable a heterogeneous phenomenon that involves different neuronal networks in different regions of an epileptic brain,” and depending on in which specific areas they arise, they can interfere with ictal events, both leading to or preventing an ictal discharge [38]. 

Some authors have searched for a relationship between the location of seizure onset and the morphological seizure pattern, dividing cases into mesial frontal and lateral frontal [25]. In this regard, no studies have been found that correlate electrode location with the morphological pattern at onset in FLE. In temporal lobe epilepsy, Ebersole et al. have found a relationship between the morphological pattern at seizure onset and the location of onset [37]. In this study IED just prior to ictal onset was easily detected but very difficult to link to a location, as the expression was widespread. Regarding the other morphological patterns at onset, the seizures arising from prefrontal electrodes did not present with a global attenuation pattern. Simultaneously, the global attenuation pattern was the most commonly seen in the seizures with undetermined onset location.

### Limitations

In this study, some temporal lobe epilepsies could have been selected, constituting a selection bias. The focus on maximal amplitude in inferior frontal regions IED (anterior temporal F7-F8) led to cases being included only if mid temporal amplitude of IED was lower than in other frontal regions. Regarding ictal activity, none of the patients had extra-frontal electrodes ictal onset or initial ictal temporal clinical signs. Still, it cannot be certain whether all cases of temporal lobe epilepsy have been excluded by this criterion and, at the same time, being too strict, only a representation of the typical electrical expression situation could have been selected, omitting some cases of frontal lobe epilepsy. This limitation is inherent in the surface EEG technique.

This study was performed on adults. Studies in children would help to see whether these results apply to frontal lobe epilepsy expression in that population.

EEG recordings registered from both analog and digital systems have been studied. Analogical recordings lack the possibility of changing montages for the EEG reading, which has limited the possibility of assigning an electrode for ictal onset in some of the recordings.

## 5. Conclusions

According to our data, isolated sharp waves, both with simple and dysphasic morphology, are the most frequent waveforms in the expression of frontal lobe IED, especially with a voltage of 50−100 µV. Spikes usually appear isolated as interictal epileptiform discharges, with a voltage of less than 50 µV. To help in the IED location, it will be necessary on many occasions to look for epochs in which the discharges present as being less active. In this context, the recording of REM sleep can be useful to locate the focus. 

Regarding the seizure onset, four possible EEG categories for seizure onset pattern were observed: rhythmic ictal discharge of >20 µV, low amplitude ictal beta rhythm (<20 µV), global attenuation (<10 µV), and IED just prior to the ictal onset accounting for 41.17%, 27.45%, 19.60% and 11.76%, respectively, in our series. Frontal lobe seizures are a diagnostic challenge, especially due to the short duration, the low amplitude seizure onset, and the subtle semiology of some frontal seizures.

Frequently the ictal expression of the electrical activity in frontal lobe seizure is subtle and challenging to interpret. Postictal activity, polygraphy changes, and the use of different EEG montages are useful for locating and identifying ictal activity in FLE.

Good quality EEG recordings, with continuous technique supervision and the best signal/noise ratio, is mandatory to perform good EEG readings in these cases [39].

The summary of the EEG observations of frontal seizure onset described in these series can be useful in the daily routine to help identify frontal seizures.

## Figures and Tables

**Table 1:** Distribution of seizure onset location.

Pre-Frontal		Central-Frontal		Inferior-Frontal		Midline-Frontal	Defined by Side		Undetermined
Fp1	Fp2	F3	F4	F7	F8	Fz	Left Unspecific	Right Unspecific	
5	4	6	4	8	5	1	5	5	8
